# Overexpression of β-Ketoacyl-CoA Synthase From *Vitis vinifera* L. Improves Salt Tolerance in *Arabidopsis thaliana*

**DOI:** 10.3389/fpls.2020.564385

**Published:** 2020-11-12

**Authors:** Zhen Yang, Xue Yang, Shujia Dong, Yao Ge, Xuenan Zhang, Xinjie Zhao, Ning Han

**Affiliations:** ^1^Shandong Provincial Key Laboratory of Microbial Engineering, School of Biologic Engineering, Qilu University of Technology (Shandong Academy of Sciences), Jinan, China; ^2^State Key Laboratory of Crop Biology, College of Life Science, Shandong Agricultural University, Tai’an, China; ^3^School of Biology and Food Engineering, Bozhou University, Bozhou, China

**Keywords:** *VvKCS11*, suberin lamellae, salt stress, *Vitis vinifera* L., salt tolerance

## Abstract

Grape (*Vitis vinifera* L.) is a fruit tree with high salt tolerance and high nutritional value, medicinal value, and economic value. Suberin in roots is characterized by long-chain fatty acids and is thought to be related to the salt tolerance of grape. The key enzyme in the fatty acid elongation process is β-ketoacyl-CoA synthase (KCS). The function and the regulatory mechanism of *VvKCS* in response to salt stress in grape are unclear. In this study, *VvKCS* was isolated from *V. vinifera* L. A real-time quantitative polymerase chain reaction analysis showed that salt stress enhanced *VvKCS* transcription levels in grapes. Overexpression of *VvKCS* increased the tolerance to salt stress in *Arabidopsis* during the germination and seedling stages. The improved salt tolerance was the result of the combined contributions of multiple mechanisms including the regulation of expression of ion transporters and channels, accumulation of osmotic regulating substances, and maintenance of membrane stability. The results of this study are valuable information on plant salt tolerance and provide a theoretical basis for the molecular mechanism of grape salt tolerance.

## Introduction

Soil salinization is a global ecological problem. Salt stress is not only a major abiotic stress in agriculture development but is also a major factor causing environmental deterioration worldwide (Munns and Tester, 2008; Yao et al., 2013). Identifying of salt-tolerance-related genes and cultivating of salt-tolerant plants are regarded as the most effective ways to improve and utilize salty land.

Grape (*Vitis vinifera* L.) is a fruit with high nutritional value, medicinal, and economic value. It is used as fresh food, and to prepare raisins, wine, and various health products. Among fruit species widely grown around the world, grape shows a relatively higher tolerance to salt stress (Daldoul et al., 2008; Walker et al., 2010). The salt tolerance of many species is characterized by lower Na^+^ content in leaves and higher Na^+^ content in roots. This salt exclusion characteristic of the plant is helpful in resisting salt stress in grape (Bing et al., 2006; Mozafari et al., 2018). Plants have several ways to exclude salt. (A) The root apoplast barriers are composed of the casparian bands (CB) and the suberin lamellae (SL), which can block ions that transport to the shoots (Pollard et al., 2008; Krishnamurthy et al., 2011). The most efficient pathway for water and solutes to enter to the shoots is apoplast transpiration bypass flow (Chloé et al., 2019). (B) Na^+^ that has been transported into the xylem can be transported back into the xylem parenchyma cells. Then, the high-affinity K^+^ transporter (HKT) catalyzes the efflux of Na^+^ through the cortex to the epidermis (Byrt et al., 2007). (C) Additionally, Na^+^ in the epidermis can be transported back to the soil. The *SOS1* gene encodes a plasma membrane Na^+^/H^+^ antiporter, which catalyzes Na^+^ efflux (Shi et al., 2003).

The root endodermal cells of grapevines enhance exclusion of Na^+^ and Cl^–^ under salt stress and show greater K^+^ selectivity (Storey et al., 2010), suggesting that endodermal cells play a key role in salt exclusion in grape. The root apoplastic barriers, which is located in the endodermis, contribute to salt exclusion by blocking the transport of solutes and water. Suberin is an essential ingredient of the apoplast barrier. Suberization of the apoplast barriers has been reported under salt stress in a variety of plants (Schreiber, 2010; Mozafari et al., 2018). Therefore, the apoplast barriers in roots play an important role in protecting plants from stresses, and suberization of the apoplast barriers may enhance the survival of plants (Schreiber, 2007; Krishnamurthy et al., 2011).

Suberin is deposited in the cell walls of plants as a lipid-phenolic biopolyester (Franke et al., 2012; Nawrath et al., 2013). The suberin polymer is characterized by long-chain fatty acids (Pollard et al., 2008) and serves as a protective barrier to block uncontrolled water and solute diffusion (Hose et al., 2001; Enstone et al., 2002). It has also been demonstrated to be a stress-induced barrier (Krishnamurthy et al., 2011; Franke et al., 2012).

Two characteristic processes of suberin biosynthesis are ω-carbon oxidation and fatty acid elongation. β-Ketoacyl-CoA synthase (KCS) catalyzes the extension of fatty acid chains (Millar and Kunst, 2010). The key role of KCS is to catalyze condensation of malonyl-CoA with acyl-CoA. Hence, KCS is an important rate-limiting enzyme in the synthesis of suberin (Kolattukudy, 1981; Smirnova et al., 2013).

Many previous studies have suggested that changes in KCS encoding genes can lead to changes in suberin components and environmental responses. Serra et al. reported silencing potato *StKCS6* results in a reduction in suberin chain length (Olga et al., 2009). The abnormal lamellation (SL) of the suberin lamellae in the endodermis is observed in roots of the *Arabidopsis kcs20* and *kcs2* double mutant. Additionally, a significant reduction of C22 and C24 and an accumulation of C20 derivatives of aliphatic suberin was observed (Lee et al., 2010). The upregulation of *KCS* in algae under salt stress increased the salt tolerance of the plant (Azachi et al., 2002). *KCS1* activity also increases in response to powdery mildew fungus in barley (Chao et al., 2018). The expression of *KCS2* is induced in *Arabidopsis* under osmotic stress conditions, indicating that *KCS2* may help plants resist to drought stress (Lee et al., 2009).

Although numerous studies have investigated KCS function, the function and the molecular mechanism of *KCS* in response to salt stress in grape (*V. vinifera* L.) remain unknown. In the present study, we isolated the *VvKCS11* gene from the Crimson Seedless grape variety. Then, the *VvKCS11* gene was transformed into wild-type (WT) *Arabidopsis* to study the salt tolerance function of the *KCS* gene. Studying the mechanism of the *VvKCS* response to salt tolerance might provide a theoretical basis for plant stress resistance.

## Materials and Methods

### Plant Materials, Cultivation, and Treatment

The study was carried out using cv. Crimson Seedless and 1103P grape (*V. vinifera* L.).

The WT control was *Arabidopsis* Col-0. The Arabidopsis Biological Resource Center was the source for the *Arabidopsis* mutant *atkcs11*. The *atkcs11* (AT2G26640) homozygous mutants were verified by screening using polymerase chain reaction (PCR).

The grape stock was uniformly grown in a tissue culture bottle containing Murashige and Skoog (MS) medium and indole-3-butytric acid (0.2 mg/L) at 25 C. The stock was subcultured at 1 month intervals. Each bottle was planted with two seedlings. The salt treatment was performed after the grape tissue culture seedlings were cultured for 1 month by exposing the seedlings to 0 or 50 mM NaCl for 2 days. Then, the roots, stems, and leaves were used to determine the *VvKCS* expression pattern.

Plump seeds of the WT and mutant were selected and sterilized in by 75 and 95% ethanol for 3 and 1 min, respectively. The seeds were washed three to five times in sterilized water and were sown on half-strength MS (1/2 MS) medium with 0, 75, 100, or 125 mM NaCl. They were transferred to a culture room after 3 days of vernalization at 4 C. The length of the roots and fresh weight (FW) of the germinated seeds were measured 7 days after planting.

*Arabidopsis* seeds were planted in red square plastic pots for the adult-stage experiment. Four-leaf stage seedlings were treated with Hoagland solution for 14 days containing 0 or 100 mM NaCl.

### Expression Patterns and Gene Screening by qRT-PCR

We investigated the expression profiles of ten genes related to the synthesis and regulation of suberin in salt-tolerant Crimson Seedless and the salt-sensitive 1103P cultivars to determine the expression pattern and screen the genes related to the salt stress response. Relative expression level was determined by qRT-PCR in roots, stems, and leaves of the two grape cultivars after treatment with 0 or 50 mM NaCl for 2 days.

The primers of the ten genes were designed using Beacon Designer software (Supplementary Table 1). The internal standard gene was actin.

### *VvKCS11* Cloning and Sequencing

Total RNA was isolated from roots of Crimson Seedless and 1103P seedling using the Plant RNA Extraction Kit (Karroten). The full-length *VvKCS11* gene was determined using the National Center for Biotechnology Information reference genome. We obtained two *VvKCS11* with the forward (5′-ACGCGTCGACAGGGTTGTGGCGTTAGAG-3′) and reverse (5′-CGCGGATCCGCTAACAACCACCCTCCTC-3′) primers.

### *VvKCS11* Bioinformatics Analysis

A sequence alignment analysis was performed, the functional domains were predicted, and a phylogenetic tree was constructed using DNAstar, MegAlign, DNAman, and SMART online software, respectively.

### Transformation of *Arabidopsis*

The pRI 101 vector was constructed to form pRI 101-*VvKCS11*. Then, the *VvKCS11* gene was transformed into *Arabidopsis*. The transgenic plants were verified with the gene-specific 35S forward primer (5′-GACGCACAATCCCACTATCC-3′) and the *VvKCS11* reverse primer (5′-CGCGGATCCGCTAACAACCACCCTCCTC-3′) by PCR after screening with kanamycin. The positive overexpression lines OE2, OE3, and OE13 were randomly selected for use in subsequent experiments.

We evaluated the effects of *VvKCS11* expression in OE lines by qRT-PCR. OE line seedlings were grown on 1/2 MS medium for 7 days. The *VvKCS11* gene (forward primer: 5-AAGCAGATGGAAGATAGC-3 and reverse primer: 5′-AGTAACGAAGACAGAACCT-3′) was amplified.

### Detecting of the *KCS11 Arabidopsis* Mutant

Plants homozygous for the T-DNA insertion were selected utilizing specific primers for *atkcs11*, LP: 5′-CTTCAGACCGTCTAAACGCAG-3′, RP: 5′-CTTTTTCGCAACACTAGTGGC-3′, and T-DNA left border specific primer LBb1: 5′-ATTTTGCCGATTTCGGAAC-3′.

### Analysis of Germination Rate, Root Length, and Fresh Weight of *Arabidopsis*

The germination rate (GR), germination energy (GE), and germination index (GI) of the WT, OE lines, and mutants were measured. GR, GE, and GI were calculated using the following formulae:

$$\text{GR}=\frac{G_{7}}{T}\times 100\%$$

$$\text{GE}=\frac{\sum G_{t}}{T}\times 100\%$$

$$\text{GI}=\sum \frac{G_{t}}{D_{t}}$$

where *G*_*t*_ is the number of seeds germinated on the *t*th day, *T* is the total number of seeds, *D*_*t*_ is the number of days up to the *t*th day.

The root length and FW of the different lines were measured after 7 days.

### Determination of Na^+^ and K^+^ Content

The shoots and roots of the *Arabidopsis* lines were weighed (0.1 g). Na^+^ and K^+^ concentrations were measured by Flame photometer (Sui et al., 2015).

### Determining of Malondialdehyde Content

Leaves samples (0.1 g) were ground into a homogenate with 3 mL of 10% trichloroacetic acid. The homogenate was added to 3 mL of 0.6% thiobarbituric acid and centrifuged at 6,000 rpm for 15 min. The absorbance of the supernatant was measured at 532 and 600 nm. Malondialdehyde (MDA) (mM/g FW) = (△A × V)/(155 × W). △A = 532-600, V = Volume of supernatant, FW = Fresh weight.

### Determining of Soluble Proline Content

Leaf samples (0.1 g) were ground into a homogenate with 1.5 mL 3% aqueous sulfosalicylic acid. The solution was incubated at 100°C for 10 min and then centrifuged at 3,000 rpm for 5 min to obtain the proline extract. Finally, the reaction mixture was added into 1 mL of toluene, and the absorbance was measured at 520 nm. A standard curve was prepared. The proline content (mg g^–1^ FW) = [(mg proline mL^–1^ × mL toluene)/[(g sample)/0.1] (Abrahám et al., 2015).

### qRT-PCR of the Stress-Responsive Genes

The expression profiles of 12 salt-responsive genes in WT and transgenic *Arabidopsis* lines were evaluated by qRT-PCR. The qPCR primers are showed in Supplementary Table 2.

### Statistical Analysis

The results are from three independent experiments, and the values are presented as mean ± standard deviation. A *p* values <0.05 were considered significant.

## Results

### Expression Pattern of Suberin-Related Genes in *V. vinifera* L. in Response to Salt Stress

We determined the relative expression levels of ten suberin-related genes (*CYP86B1*, *KCS11-1*, *KCS11-2*, *KCS11-3*, *KCS4*, *CYP86A22*, *CYP86A8*, *MYB36*, *MYB39*, and *AS1*) in roots, leaves, and stalks of the Crimson Seedless and 1103P cultivars under the 0 or 50 mM NaCl treatments for 48 h. As shown in Supplementary Figure 1, *CYP86B1* and *KCS11-1* were not detected in either cultivar. Most of these genes showed higher expression level in roots of 1103P cultivar than in leaves or stalks with or without the salt treatment (Supplementary Figure 1A). However, the expression levels of these genes in different Crimson Seedless tissues were not significantly different in the two salt treatments. *KCS11-2* expression was significantly upregulated by salt stress only in roots (Supplementary Figure 1B).

### Sequence Alignment and Analysis of *VvKCS11*

The full-length sequence alignment between *VvKCS11* of Crimson Seedless and 1103P was obtained by PCR and the NCBI (Supplementary Figure 2). The gene and amino acid sequences of *VvKCS11* in Crimson Seedless were identical to those given at the NCBI. However, there were seven bases and one amino acid different in 1103P. This may be related to the difference in salt tolerance between the two cultivars, which will be further studied in the future. In the present study, we selected the *KCS11* gene of salt-tolerant Crimson Seedless for subsequent experiments to reveal the mechanism of *VvKCS11* in the salt stress response. The *VvKCS11* gene amplified from Crimson Seedless contained 547 amino acids (Supplementary Figure 3). A phylogenetic tree of the peptide sequence was constructed using the neighbor-joining method. The Crimson Seedless *VvKCS11* gene showed the highest identities with the *VvKCS11* gene from *V. vinifera* L. (Supplementary Figure 4).

### Identification of *VvKCS11* Overexpression Lines

*VvKCS11* was overexpressed in *Arabidopsis*, and T3 homozygous transgenic lines were generated (OE2, OE3, and OE13; Supplementary Figure 5).

The relative expression level of *VvKCS11* increased significantly in the *Arabidopsis* OE lines. OE2, OE3, and OE13 increased significantly (Supplementary Figure 6).

### Identification of the *atkcs11* T-DNA Insertion Mutant

The *atkcs11* T-DNA insertion mutants were identified by PCR analysis. Lanes 1–5 represent homozygous *Arabidopsis* mutant *kcs11*. *kcs11* was expressed at low levels in the mutant *Arabidopsis* lines (Supplementary Figure 7), indicating that *Arabidopsis* mutant *atkcs11* has been showed a homozygous mutant in AT2G26640.

### The Phenotype, Fresh Weight, and Root Length of Different *Arabidopsis* Lines Treated With Different Salt Concentrations at the Germination Stage

**FIGURE 1:**
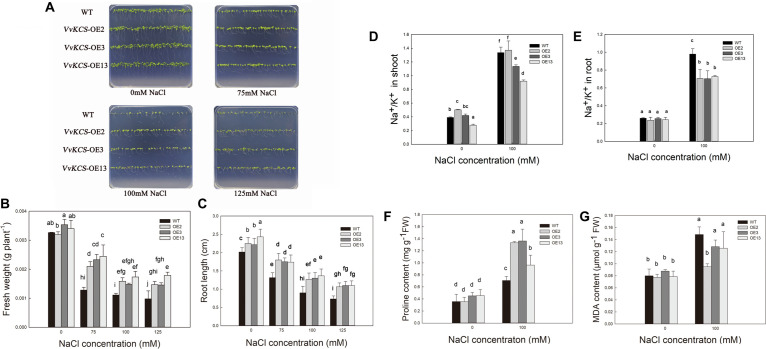
The phenotype **(A)**, fresh weight **(B)**, root length **(C)**, Na^+^/K^+^ ratio in shoot **(D)**, and root **(E)**, proline content **(F)**, and MDA content **(G)** of the WT and transgenic *Arabidopsis* plants lines under different NaCl concentrations for 7 days.

As shown in Figure 1, no significant differences were found in the phenotype, FW or root length (RL) of the WT and *Arabidopsis* OE lines under the control condition. Under the different NaCl treatments, FW and RL of the WT and overexpressed *Arabidopsis* lines were inhibited, which were more severe in WT, particularly under the higher NaCl concentration (100 and 125 mM; Figure 1A). FW of the WT, OE2, OE3, and OE13 decreased by 61, 34, 34, and 28%, respectively, in the 75 mM NaCl treatment. FW of WT, OE2, OE3, and OE13 plants decreased by 66, 50, 58, and 49%, respectively, after being treated with 100 mM NaCl. FW of the WT, OE2, OE3, and OE13 plants decreased by 70, 54, 59, and 47%, respectively, in the 125 mM NaCl treatments (Figure 1B). The RL of the WT, OE2, OE3, and OE13 decreased by 35, 20, 21, and 28%, respectively, in the 75 mM NaCl treatment. The RL of the WT, OE2, OE3, and OE13 decreased by 55, 44, 41, and 44%, respectively, in the 100 mM NaCl treatment. The RL of the WT, OE2, OE3, and OE13 decreased by 64, 53, 50, and 55%, respectively, in the 125 mM NaCl treatment (Figure 1C). Hence, overexpression of *VvKCS11* enhanced the growth of *Arabidopsis* during the germination stage under salt stress.

### Germination Percentages of the Different *Arabidopsis* Lines Under Salt Stress

The germination rates of the WT and the *VvKCS11* OE lines were not different under the control condition. The germination indicators of the different *Arabidopsis* lines were inhibited at 75 mM NaCl, particularly the GI. In contrast, the overexpressed lines maintained relatively higher levels of the germination indicators. After treatment, the GI of OE2, OE3, and OE13 decreased by 66, 20, 19, and 28%, respectively. In addition, the GR of OE2, OE3, and OE13 decreased by1.08, 1.2, and 0.9 times of the WT in the 125 mM NaCl treatment, respectively. These results indicate that overexpression *KCS11* could protect plants from salt stress during the germination stage (Supplementary Figure 8).

### The Na^+^/K^+^ Ratio in the Different *Arabidopsis* Lines Under Salt Stress at the Germination Stage

The Na^+^/K^+^ ratio in the OE lines and WT plants was not significantly different under the control condition (Figures 1D,E). Na^+^/K^+^ trended to increase in all lines under the salt treatments. However, Na^+^/K^+^ in the three OE lines was lower than that in the WT. Hence, overexpression *KCS11* could protect plants from salt stress during the germination stage (Figures 1D,E).

### Changes in Proline and MDA Content in the Different *Arabidopsis* Lines Under Salt Stress at the Germination Stage

Proline content was not significantly different between the WT and OE lines in the control group (Figure 1F). Proline content increased after the salt treatment. Proline content of the WT, OE2, OE3, and OE13 increased by 98, 278, 202, and 111%, respectively (Figure 1F).

MDA content did not change in any of the *Arabidopsis* lines in the control group (Figure 1G). The MDA content of the WT, OE2, OE3, and OE13 increased by 85, 23, 46, and 60%, respectively, in NaCl treatment (Figure 1G).

### The Phenotype and Fresh Weight in the Different *Arabidopsis* Lines Under Salt Stress at the Seedling Stage

**FIGURE 2:**
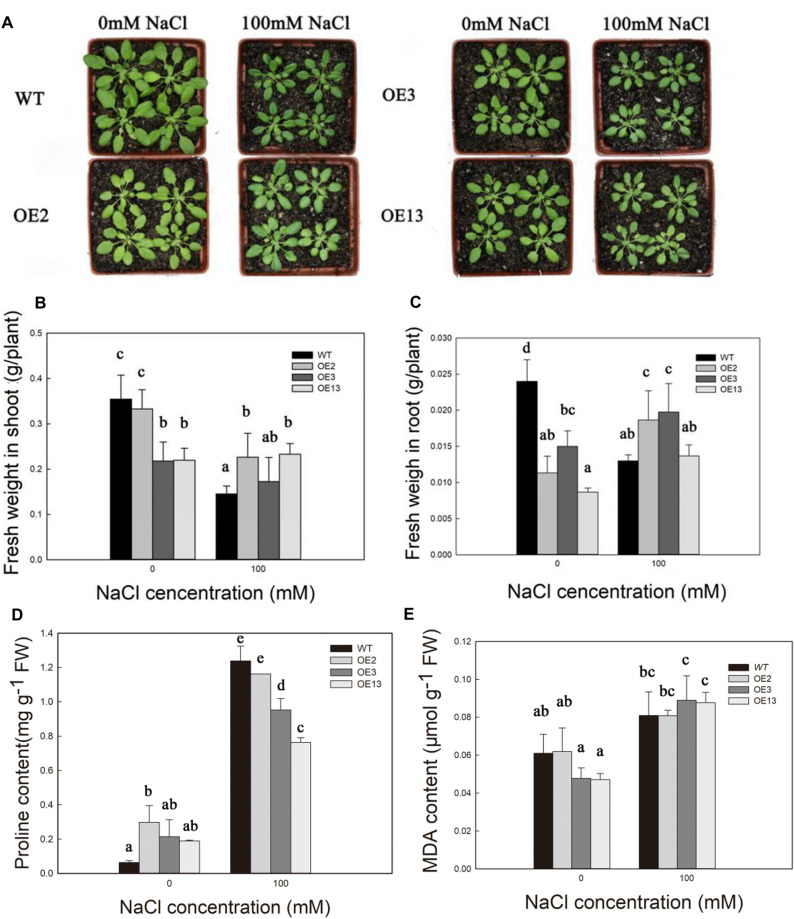
The phenotype **(A)**, and fresh weight in shoot **(B)** and root **(C)**, proline content **(D)**, and MDA content **(E)** of the WT, transgenic *Arabidopsis* plants lines under 0 and 100 mM NaCl concentrations for 14 days.

As shown in Figure 2, the WT and OE *Arabidopsis* lines grew well overall in the control group. Growth of the *Arabidopsis* lines was inhibited after the salt treatment. However, the phenotype of the OE lines was less suppressed than that of WT (Figure 2A).

The FW of the shoots of the WT, OE2, and OE3 *Arabidopsis* lines decreased by 59, 32, and 21%, respectively, in the 100 mM salt treatment. However, OE13 shoot FW increased by 6%. FW of WT roots decreased by 46% under salt stress, while FW of roots in OE2, OE3, and OE13 increased by 65, 32, and 58%, respectively (Figures 2B,C).

### Changes in Proline and MDA Contents in Different *Arabidopsis* Lines Under Salt Stress at the Seedling Stage

The proline content was not different between the WT and OE lines in the control group (Figure 2D). The proline content of the WT, OE2, OE3, and OE13 increased by 190, 290, 345, and 556%, respectively, after treatment with 100 mM NaCl (Figure 2D).

MDA content was relatively low in all the *Arabidopsis* lines in the control group (Supplementary Figure 10B). The MDA increased but was not significantly different between the *Arabidopsis* lines under any of the NaCl treatments (Figure 2E).

### The Phenotype, Fresh Weight, and Root Length in WT and *atkcs11* Under Salt Stress at the Germination Stage

**FIGURE 3:**
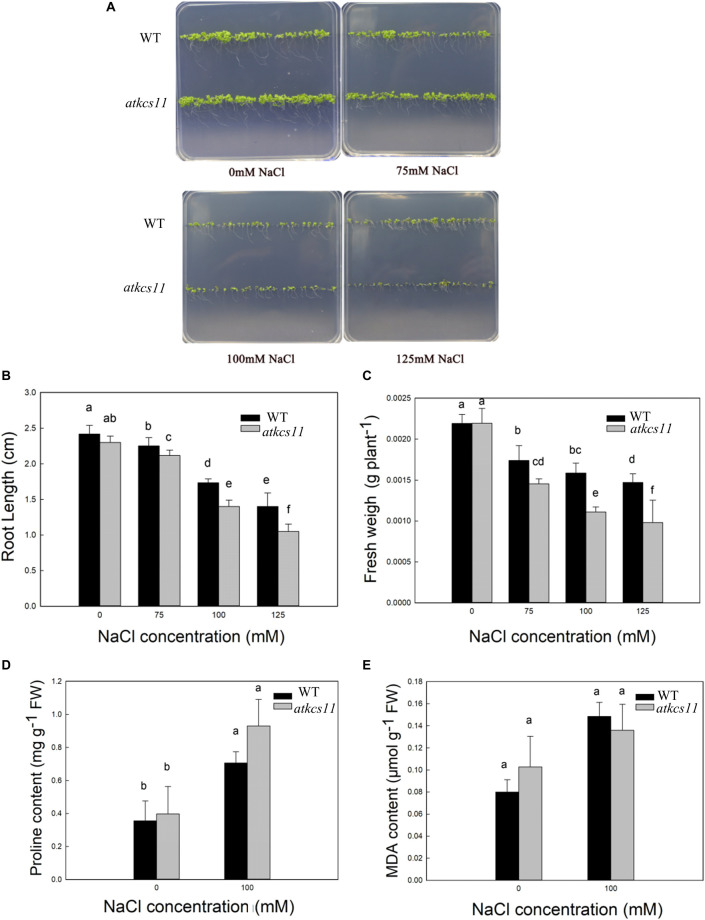
The phenotype **(A)**, root length **(B)**, fresh weight **(C)**, proline content **(D)**, and MDA content **(E)** in the WT and T-DNA mutant *Arabidopsis* lines under different NaCl concentrations for 7 days.

The phenotypes of the WT and *atkcs11* in the control group were not different. However, the growth rates of the WT and mutant were affected by salt stress (Figure 3A). The root length of the WT and *atkcs11* decreased by 28 and 39%, respectively, after treatment with 100 mM NaCl. FW of the WT and *atkcs11* decreased by 28 and 38%, respectively (Figure 3B). The root length of the WT and *atkcs11* plants decreased by 42 and 54%, respectively, under the 125 mM NaCl treatment (Figure 3C). The FW of the WT and *atkcs11* decreased by 28 and 38%, respectively, after treatment with 100 mM NaCl. FW of the WT and *atkcs11* plants decreased by 35 and 46%, respectively (Figure 3C).

### Changes in Proline and MDA Content in WT and *atkcs11* Under Salt Stress at the Germination Stage

The proline content observed in the WT and T-DNA mutant *Arabidopsis* lines was not significantly different in the control group (Figure 3D). However, the proline content of the different *Arabidopsis* lines increased, and the proline content of the WT and *atkcs11* increased by 98 and 134% in the salt treatment groups (Figure 3D).

MDA is a product of membrane peroxidation. MDA content was not different in the WT or*atkcs11* from the control group (Figure 3E). Under NaCl treatment, the MDA content of the WT and *atkcs11* increased by 85 and 32% under the NaCl treatment (Figure 3E).

### The Phenotype and Fresh Weight in the WT and T-DNA Mutant *Arabidopsis* Lines Under Salt Stress at the Seedling Stage

**FIGURE 4:**
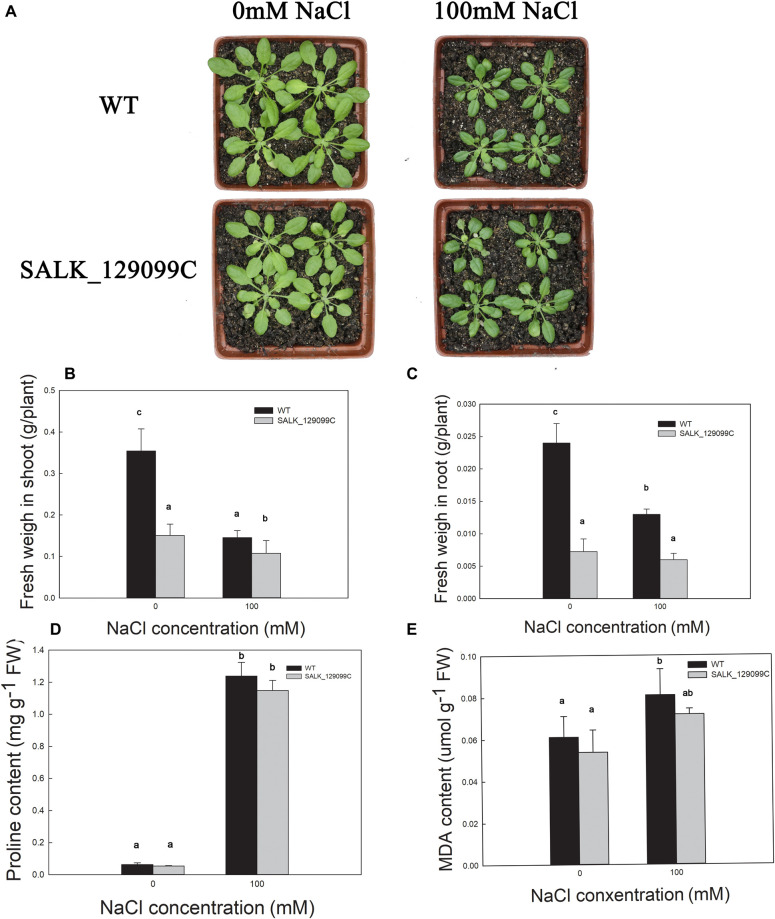
The phenotype **(A)**, and fresh weight in shoot **(B)** and root **(C)**, proline content **(D)**, MDA content **(E)** of the WT and T-DNA mutant *Arabidopsis* lines treated with 0 and 100 mM NaCl for 14 days.

As shown in Figure 4A, the WT and T-DNA mutant *Arabidopsis* lines grew well in the control group. However, the growth of the different *Arabidopsis* lines was inhibited by the salt treatment. However, the phenotype of *atkcs11* was more suppressed than that of WT (Figure 4A).

FW of the shoots of the WT and *atkcs11* decreased by 59 and 28% (Figure 4B), and the FW of roots in the WT and *atkcs11 Arabidopsis* lines increased by 45 and 17% under 100 mM salt treatment (Figure 4C).

### Changes in Proline and MDA Content in the WT and *atkcs11* Under Salt Stress at the Seedling Stage

Proline content was not different between the WT and *atkcs11* in the control group (Figure 4D). Proline content in the WT and *atkcs11* increased by 198 and 109%, respectively, in response to the salt treatment (Figure 4D).

Additionally, MDA content was not different between the WT and *atkcs11* in the control group (Figure 4E). MDA content increased significantly in the WT and *atkcs11* after treatment with 100 mM NaCl, but no significant difference was observed between them (Figure 4E).

### Expression of Ion Transport-Related Genes in *VvKCS11* Transgenic Arabidopsis Plants

**FIGURE 5:**
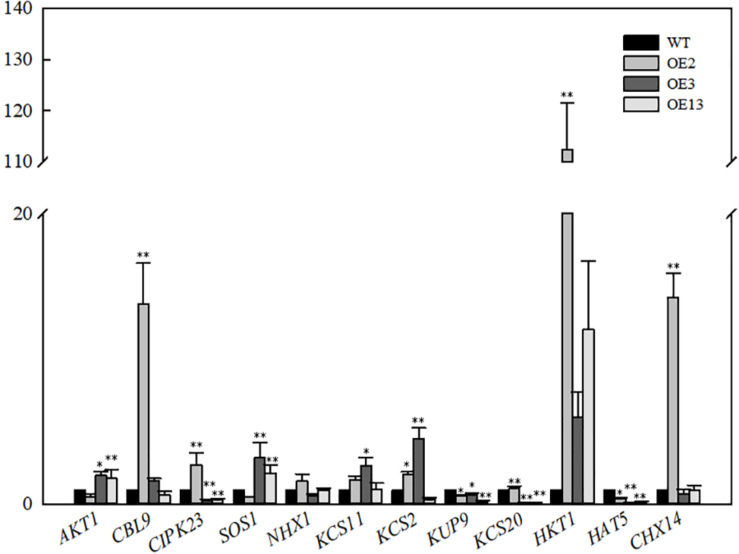
Relative expression levels of genes related ion transport in *VvKCS11* transgenic *Arabidopsis* plants.

The relative expression levels of genes related to 12 ion transporters were determined in the transgenic lines and WT. As shown in Figure 5, the *AKT1* expression level in OE3 and OE13 was higher than that in the WT. *AKT1* expression was slightly lower in OE2 than that in the WT. However, the expression of *CBLl9* and *CIPK23* was significantly upregulated. The SOS1 result was similar to that of AKT1. The expression level of SOS1 in OE3 and OE13 was upregulated and no significant change was observed in OE2. Additionally, OE2, OE3, and OE13 exhibited high expression levels of *HKT1* compared with WT.

## Discussion

Grape (*V. vinifera* L.) is an important agricultural product worldwide, which is mainly used as a fresh food or in wine, raisins, and health products (Mnari et al., 2016; Colombo et al., 2019). Some vineyards are located in semiarid areas accompanied by soil salinization (Benjak et al., 2005). Grape is relatively tolerant to salinity (Daldoul et al., 2010; Mozafari et al., 2018). Salt tolerance is correlated with salt exclusion, which limits the transportation of Na^+^ from the roots to the shoots (Bernstein, 1977). Salt exclusion is an important way for grapes to resist salt stress (Bernstein, 1980).

The root apoplastic barriers are composed of Casparian band and suberin lamella, and they play an important role in blocking apoplastic bypass flow of water and ions into the stele and Na^+^ transport into the shoots. The root apoplastic barriers are strengthened by stress (Krishnamurthy et al., 2011; Vishal et al., 2019). Additionally, it has been reported that the suberin content of salt-tolerant plant species increases in response to salt stress (Beisson et al., 2007; Krishnamurthy et al., 2009).

In plants, the function of the SL in blocking water and ions is mainly dependent on aliphatic domains (Bernards, 2002). The main components of the suberin aliphatic domains are long chain ω-hydroxy fatty acids (Gandini et al., 2006). The main function of KCS is to catalyze acyl chain elongation for fatty acid chain elongation (Beisson et al., 2007; Lee et al., 2009). Some studies have shown that the expression of *KCS* is upregulated under salt stress (Azachi et al., 2002).

In the present study, many genes were upregulated in 1103P and Crimson Seedless under salt stress (Supplementary Figure 1). *CYP86B1*, *KCS11-1*, *KCS11-2*, *KCS11-3*, *KCS4*, *CYP86A22*, and *CYP86A8* are genes associated with suberin precursor synthesis. *MYB36* and *MYB39* participated in the regulation of suberin synthesis. The transcription factor *AS1* promotes gene expression. Some showed root-specific expression trends, which may be related to formation of the root apoplastic barriers. However, the expression patterns of these genes were different in the two varieties. In the salt-sensitive 1103P, these genes were expressed at higher levels in the control group and were slightly upregulated after salt stress (Supplementary Figure 1A). In the salt-tolerant Crimson Seedless, the expression level of the control group was very low and extremely enhanced by the salt stress (Supplementary Figure 1B). These results indicate that the VLCFA synthesized by *VvKCS11* may play different roles in the two grape varieties. In 1103P, *VvKCS11* had a high background expression level under the control treatment. The VLCFA may mainly participate in other biosynthetic or metabolic processes rather than the formation of the SL. However, the background expression level of *VvKCS11* was much lower in Crimson Seedless. In addition, the expression level was significantly induced by salt stress. Salt stress was a direct inducer of *VvKCS11* in Crimson Seedless, suggesting that the VLCFA catalyzed by *KCS11* may be involved in the salt stress response, such as participation in the formation of SL in roots. Most studies of this gene have focused on plant development, so little is known about its function in the salt response. In order to reveal the role of *VvKCS11* in response to salt stress, we screened and identified heterologous OE lines of *VvKCS11* in *Arabidopsis* (OE2, OE3, and OE13) and a T-DNA insertion mutant (Supplementary Figure 7).

Studying the effect of salt stress on seed germination is of great significance (Atská, 1976). In the present study, we determined that the GR, FW, and root length of all lines were inhibited in the NaCl-treated groups. The degree of inhibition in the WT was more severe than that in the OE lines but slightly less than that of the mutant, indicating that overexpression of *VvKCS11* may increase salt tolerance. We obtained similar results in the seedling stage experiments to those in the germination stage. The phenotype and FW of shoots and roots in all lines were inhibited under the 100 mM NaCl treatment. The degree of inhibition in the WT was the highest showing that that overexpression of *VvKCS11* may help the plants increase salt tolerance.

The increased Na^+^ content in plant cells during salt stress has been well established (Rus et al., 2001). Salt stress destroys the permeability of cell membranes. Hence, the ionic balance of cells is destroyed (Amar et al., 2000; Tester and Davenport, 2003). Therefore, the Na^+^/K^+^ ratio is an important indicator of the ability of plants to maintain their ion balance and salt tolerance (Wang et al., 2020). In the present study, the Na^+^/K^+^ ratio increased in the WT and OE lines, but the increases were much lower in the OE lines, suggesting that overexpression *VvKCS11* may lead to a stronger control of ionic homeostasis in response to salt stress. Ions are taken up by ion channels or transporters on the plasma membrane. Overexpression of the *VvKCS11* gene may play a role in regulating the expression of these ion transport-related genes.

The thickened SL mat that developed after the salt treatment and overexpression of the suberin-related gene *VvKCS11* effectively blocked the water and ions entering the plant through the apoplastic pathway. This ensures that the Na^+^ concentration in the plant is maintained at a relatively lower level. However, the SL is not selective in blocking ions, as it also blocks beneficial ions necessary for plant growth. Thus, the genes involved in ion transport need to be regulated to ensure the growth of the plant. According to our results, the genes related to ion transport changed in the OE lines.

The expression levels of *AKT1* in OE3 and OE13 were higher than that in the WT, indicating that the overexpression of *VvKCS11* induced expression of the K^+^ channel protein, which increases K^+^ under salt stress. Calcineurin B-like proteins (CBL) and CBL-interacting protein kinases (CIPK) have been proven to mediate plant responses to a variety of external stressors. Although much effort has gone into understanding the role played by CIPKs in the response to stress, the functions of only a few CIPKs are clear. CIPK23 maintains ion homeostasis under salt stress (Luan, 2009). The CBL9-CIPK23 complex is required to activate the *Arabidopsis* K*^+^* transporter 1 (AKT1) channel (Li et al., 2006; Xu et al., 2006). Although the expression level of *AKT1*in OE2 was slightly lower than that in the WT, the expression levels of *CBLl9* and *CIPK23* were significantly upregulated. This indicates that overexpression of *VvKCS11* could induce activation of the K^+^ channel protein in the OE2 line, which would increase absorption of K^+^.

OE2, OE3, and OE13 showed high expression levels of *HKT1* compared with the WT indicating that overexpression of *VvKCS11* induced the expression and function of *HKT1*, which may further promote unloading of Na^+^ from aboveground parts and transport to underground parts, leading to relatively lower Na^+^ content in the aboveground parts. In addition, the expression levels of *SOS1* in OE3 and OE13 were upregulated. Na^+^ transported underground by HKT1 can be transported out through the SOS1 Na^+^/H^+^ antiporter. The *SOS1* expression level in OE2 did not change significantly, which may be related to the difference in *VvKCS11* expression and the insertion site among the three lines.

Plants under stress adapt to the environment by accumulating compatible osmolytes (Dreier, 1983; Ábrahám et al., 2003). Proline acts as important osmolyte that accumulates in plants under stress conditions (Rejeb et al., 2015). Additionally, proline plays an important role stabilizing the structure of macromolecules and regulating the cellular redox potential (Szekely et al., 2010). Proline content increased under salt stress in all lines and that the highest is in the OE lines. These results showed that overexpressing*VvKCS11* may increase the osmotic adjustment rate in *Arabidopsis*.

NaCl stress leads to the accumulation of MDA, an end-product of membrane peroxidation (Li et al., 2012; Wang et al., 2019). Therefore, lower MDA content may reflect stronger resistance to salt stress (Zahra et al., 2017). In the present study, MDA content increased under salt stress. The MDA content was lower in the OE lines. These findings are consistent with those of Guo et al. and suggest that overexpression *VvKCS11* may play a key role in salt stress.

In conclusion, we demonstrated that the expression of *KCS* in Crimson Seedless was activated by salt stress. Overexpression of *VvKCS11* increased the tolerance to salt stress in *Arabidopsis* during the germination and seedling stages. The improved salt tolerance was the result of the combined contributions of multiple mechanisms, including the regulation of ion transporters and channels, accumulation of osmoregulating substances, and maintenance of membrane stability. This study provides a theoretical basis to study resistance to stress in plants.

## Data Availability Statement

The datasets presented in this study can be found in online repositories. The names of the repository/repositories and accession number(s) can be found in the article/ Supplementary Material.

## Author Contributions

XY performed most of the experiments. ZY did part of the research, drafted the manuscript. XY, SD, YG, X-NZ, and X-JZ collected data and carried out all analysis. NH conceptualized the idea and revised the manuscript. All authors contributed to the article and approved the submitted version.

## Conflict of Interest

The authors declare that the research was conducted in the absence of any commercial or financial relationships that could be construed as a potential conflict of interest. The reviewer NS declared a past co-authorship with one of the authors, ZY, to the handling editor.

## References

[B1] Abrahám E. Hourton-Cabassa C. Erdei L. Szabados L. (2015). Methods for determination of proline in plants. *Methods Mol. Biol.* 639 317–331. 10.1007/978-1-60761-702-0_20 20387056

[B2] Ábrahám E. Rigó G. Székely G. Nagy R. Koncz C. Szabados L. (2003). Light-dependent induction of proline biosynthesis by abscisic acid and salt stress is inhibited by brassinosteroid in *Arabidopsis*. *Plant Mol. Biol.* 51 363–372. 10.1023/A:1022043000516 12602867

[B3] Amar N. Messenguy F. Bakkoury M. E. Dubois E. (2000). ArgRII, a component of the ArgR-Mcm1 complex involved in the control of arginine metabolism in saccharomyces cerevisiae, is the sensor of arginine. *Mol. Cell Biol.* 20 2087–2097. 10.1128/MCB.20.6.2087-2097.2000 10688655 PMC110825

[B4] Atská V. (1976). The germination of seeds. *Biol. Plant.* 18 220–220. 10.1007/BF02922809

[B5] Azachi M. Sadka A. Fisher M. Goldshlag P. Gokhman I. Zamir A. (2002). Salt induction of fatty acid elongase and membrane lipid modifications in the extreme halotolerant alga *Dunaliella salina*. *Plant Physiol.* 129 1320–1329. 10.1104/pp.001909 12114585 PMC166525

[B6] Beisson F. Li Y. Bonaventure G. Mike P. Ohlrogge J. B. (2007). The acyltransferase GPAT5 is required for the synthesis of suberin in seed coat and root of *Arabidopsis*. *Plant Cell* 19 351–368. 10.1105/tpc.106.048033 17259262 PMC1820950

[B7] Benjak A. Ercisli S. Vokurka A. Maletic E. Pejic I. (2005). Genetic relationships among grapevine cultivars native to Croatia, Greece and Turkey. *Vitis* 44 73–77. 10.1007/s00122-004-1850-2 15580473

[B8] Bernards M. A. (2002). Demystifying suberin [Review]. *Can. J. Bot.* 80 227–240. 10.1016/j.canlet.2009.01.019 19217205

[B9] Bernstein L. (1977). Physiological basis of salt tolerance in plants. *Basic Life Ences* 8 283–290. 10.2136/sssaj1965.03615995002900020003x 23091

[B10] Bernstein L. (1980). Salt tolerance of fruit crops. *US Department Agr Info Bull.* 292 1–8. 10.1016/s0378-3774(00)00099-8

[B11] Bing Z. Y. Huai L. C. Sheng S. H. Wang M. F. (2006). Relation between salt tolerance of grape rootstock and MDA and proline contents in grape leaves. *Acta Bot. Boreali Occidentalia Sin.* 18 315–319. 10.5207/JIEIE.2006.20.9.032

[B12] Byrt C. S. Platten J. D. Spielmeyer W. James R. A. Lagudah E. S. Dennis E. S. (2007). HKT1;5-like cation transporters linked to Na^+^ exclusion loci in wheat, NAX2 and KNA1. *Plant Physiol. Plant Physiol.* 143 1918–1928. 10.1104/pp.106.093476 17322337 PMC1851807

[B13] Chao L. Haslam T. M. Anna K. Schneider L. M. Kohei M. Lacey S. (2018). The β-Ketoacyl-CoA synthase HvKCS1, encoded by Cer-zh, plays a key role in synthesis of barley leaf wax and germination of barley powdery mildew. *Plant Cell Physiol.* 59:4. 10.1093/pcp/pcy020 29401261

[B14] Chloé C. Jorge B. Marie B. Valérie R. Christophe M. (2019). Regulation of a plant aquaporin by a Casparian strip membrane domain protein-like. *Plant Cell Environ.* 42 1788–1801. 10.1111/pce.13537 30767240

[B15] Colombo F. Di Lorenzo C. D. L. Regazzoni L. Fumagalli M. Sangiovanni E. de Sousa L. P. (2019). Phenolic profile and anti-inflammatory activity of sixteen Grape (*Vitis vinifera* L.) varieties. *Food Funct.* 10 1797–1807. 10.1039/c8fo02175a 30778463

[B16] Daldoul S. Guillaumie S. Reustle G. T. M. Krczal G. Ghorbel A. Delrot S. (2010). Isolation and expression analysis of salt induced genes from contrasting grapevine (*Vitis vinifera* L.) cultivars. *Plantence* 179 489–498. 10.1016/j.plantsci.2010.07.017 21802607

[B17] Daldoul S. Höfer M. U. Linhard C. Jellouli N. Mliki A. Reustle G. M. (2008). “Expression analysis of salt stress responsive genes in grapevines,” in *Biosaline Agriculture and High Salinity Tolerance*, eds Abdelly C. Öztürk M. Ashraf M. Grignon C. (Birkhäuser Basel), 297–303. 10.1007/978-3-7643-8554-5_27

[B18] Dreier W. (1983). The content of proline and the salt resistance of plants. *Biol Plant.* 25:81 10.1007/BF02902112

[B19] Enstone D. E. Peterson C. A. Ma F. (2002). Root endodermis and exodermis: structure, function, and responses to the environment. *J. Plant Growth Regul.* 21 335–351. 10.1007/s00344-003-0002-2

[B20] Franke R. B. Dombrink I. Schreiber L. (2012). Suberin goes genomics: use of a short living plant to investigate a long lasting polymer. *Front. Plant Ence* 3:4. 10.3389/fpls.2012.00004 22639633 PMC3355613

[B21] Gandini A. Neto C. P. Silvestre A. J. D. (2006). Suberin: a promising renewable resource for novel macromolecular materials. *Prog. Polym. Ence* 31 878–892. 10.1016/j.progpolymsci.2006.07.004

[B22] Hose E. Clarkson D. T. Steudle E. Schreiber L. Hartung W. (2001). The exodermis: a variable apoplastic barrier. *J. Exp. Bot.* 52 2245–2264. 10.1093/jexbot/52.365.2245 11709575

[B23] Kolattukudy P. (1981). Structure, biosynthesis, and biodegradation of cutin and suberin. *Annu. Rev. Plant Physiol.* 32 539–567. 10.1146/annurev.pp.32.060181.002543

[B24] Krishnamurthy P. Ranathunge K. Franke R. Prakash H. S. Schreiber L. Mathew M. K. (2009). The role of root apoplastic transport barriers in salt tolerance of rice (*Oryza sativa* L.). *Planta* 230 119–134. 10.1007/s00425-009-0930-6 19363620

[B25] Krishnamurthy P. Ranathunge K. Nayak S. Schreiber L. Mathew M. K. (2011). Root apoplastic barriers block Na+ transport to shoots in rice (*Oryza sativa* L.). *J. Exp. Bot.* 62 4215–4228. 10.1093/jxb/err135 21558150 PMC3153681

[B26] Lee S. B. Jung S. J. Go Y. S. Kim H. U. Kim J. K. Cho H. J. (2009). Two *Arabidopsis* 3-ketoacyl CoA synthase genes, KCS20 and KCS2/DAISY, are functionally redundant in cuticular wax and root suberin biosynthesis, but differentially controlled by osmotic stress. *Plant J.* 60 462–475. 10.1111/j.1365-313x.2009.03973.x 19619160

[B27] Lee S. B. Jung S. J. Go Y. S. Kim H. U. Kim J. K. Cho H. J. (2010). Two *Arabidopsis* 3-ketoacyl CoA synthase genes, KCS20 and KCS2/DAISY, are functionally redundant in cuticular wax and root suberin biosynthesis, but differentially controlled by osmotic stress. *Plant J.* 60 462–475. 10.1111/j.1365-313X.2009.03973.x 19619160

[B28] Li F. Wu Q. Y. Duan M. Dong X. C. Li B. Meng Q. W. (2012). Transgenic tomato plants overexpressing chloroplastic monodehydroascorbate reductase are resistant to salt- and PEG-induced osmotic stress. *Photosynthetica* 50 120–128. 10.1007/s11099-012-0021-y

[B29] Li L. Kim B. Cheong Y. Pandey G. Luan S. (2006). A Ca^2 +^ signaling pathway regulates a K^+^ channel for low-K response in *Arabidopsis*. *Proc Natl Acad Sci U.S.A.* 103 12625–12630. 10.1073/pnas.0605129103 16895985 PMC1567929

[B30] Luan S. (2009). The CBL-CIPK network in plant calcium signaling. *Trends Plant Ence* 14 37–42. 10.1016/j.tplants.2008.10.005 19054707

[B31] Millar A. A. Kunst L. (2010). Very-long-chain fatty acid biosynthesis is controlled through the expression and specificity of the condensing enzyme. *Plant J.* 12 121–131. 10.1046/j.1365-313X.1997.12010121.x 9263455

[B32] Mnari A. B. Harzallah A. Amri Z. Aguir S. D. Hammami M. (2016). Phytochemical content, antioxidant properties, and phenolic profile of Tunisian Raisin Varieties (*Vitis Vinifera* L.). *Int. J. Food Prop.* 19 578–590. 10.1080/10942912.2015.1038720

[B33] Mozafari A. A. Ghadakchi Asl A. Ghaderi N. (2018). Grape response to salinity stress and role of iron nanoparticle and potassium silicate to mitigate salt induced damage under in vitro conditions. *Physiol. Mol. Biol. Plants* 24 25–35. 10.1007/s12298-017-0488-x 29398836 PMC5787119

[B34] Munns R. Tester M. (2008). Mechanisms of salinity tolerance. *Annu. Rev. Plant Biol.* 59 651–681. 10.1146/annurev.arplant.59.032607.092911 18444910

[B35] Nawrath C. Schreiber L. Franke R. B. Geldner N. Kunst L. (2013). Apoplastic diffusion barriers in *Arabidopsis*. *Arabidopsis Book* 11:e0167. 10.1199/tab.0167 24465172 PMC3894908

[B36] Olga S. Marçal S. Carolin H. Rochus F. Lukas S. Salomé P. (2009). Silencing of StKCS6 in potato periderm leads to reduced chain lengths of suberin and wax compounds and increased peridermal transpiration. *J. Exp. Bot.* 60 697–707. 10.1093/jxb/ern314 19112170 PMC2651458

[B37] Pollard M. Beisson F. Li Y. Ohlrogge J. B. (2008). Building lipid barriers: biosynthesis of cutin and suberin. *Trends Plant.* 13 240–246. 10.1016/j.tplants.2008.03.003 18440267

[B38] Rejeb K. B. Vos D. L. D. Disquet I. L. Leprince A. S. Bordenave M. Maldiney R. (2015). Hydrogen peroxide produced by NADPH oxidases increases proline accumulation during salt or mannitol stress in *Arabidopsis thaliana*. *New Phytol.* 208 1138–1148. 10.1111/nph.13550 26180024

[B39] Rus A. Yokoi S. Sharkhuu A. Reddy M. Hasegawa P. M. (2001). AtHKT1 is a salt tolerance determinant that controls Na+ entry into plant roots. *Proc. Natl. Acad. U.S.A.* 98 14150–14155. 10.1073/pnas.241501798 11698666 PMC61183

[B40] Schreiber F. L. (2007). Suberin — a biopolyester forming apoplastic plant interfaces. *Curr. Opin. Plant Biol.* 10 252–259. 10.1016/j.pbi.2007.04.004 17434790

[B41] Schreiber L. (2010). Transport barriers made of cutin, suberin and associated waxes. *Trends Plant Sci.* 15 546–553. 10.1016/j.tplants.2010.06.004 20655799

[B42] Shi H. Lee B.-H. Wu S.-J. Zhu J.-K. (2003). Overexpression of a plasma membrane Na^+^/H^+^ antiporter gene improves salt tolerance in *Arabidopsis thaliana*. *Nat. Biotechnol.* 21 81–85. 10.1038/nbt766 12469134

[B43] Smirnova A. Leide J. Riederer M. (2013). Deficiency in a very-long-chain fatty acid β-ketoacyl-coenzyme a synthase of tomato impairs microgametogenesis and causes floral organ fusion. *Plant Physiol.* 161 196–209. 10.1104/pp.112.206656 23144186 PMC3532251

[B44] Storey R. Schachtman D. P. Thomas M. R. (2010). Root structure and cellular chloride, sodium and potassium distribution in salinized grapevines. *Plant Cell Environ.* 26 789–800. 10.1046/j.1365-3040.2003.01005.x 12803608

[B45] Sui N. Yang Z. Liu M. Wang B. (2015). Identification and transcriptomic profiling of genes involved in increasing sugar content during salt stress in sweet sorghum leaves. *BMC Genom.* 16:534. 10.1186/s12864-015-1760-5 26186930 PMC4506618

[B46] Szekely G. Abraham E. Cseplo A. Rigo G. Szabados L. (2010). Duplicated P5CS genes of *Arabidopsis* play distinct roles in stress regulation and developmental control of proline biosynthesis. *Plant J.* 53 11–28. 10.1111/j.1365-313X.2007.03318.x 17971042

[B47] Tester M. Davenport R. (2003). Na^+^ tolerance and Na^+^ transport in higher plants. *Ann. Bot.* 91 503–527. 10.1093/aob/mcg058 12646496 PMC4242248

[B48] Vishal B. Krishnamurthy P. Ramamoorthy R. Kumar P. P. (2019). OsTPS8 controls yield-related traits and confers salt stress tolerance in rice by enhancing suberin deposition. *New Phytol.* 221 1369–1386. 10.1111/nph.15464 30289560

[B49] Walker R. R. Blackmore D. H. Clingeleffer P. R. Correll R. L. (2010). Rootstock effects on salt tolerance of irrigated field-grown grapevines (*Vitis vinifera* L. cv. Sultana).: 1. Yield and vigour inter-relationships. *Aust. J. Grape Wine R.* 8 3–14. 10.1111/j.1755-0238.2002.tb00206.x

[B50] Wang P. Wang C. M. Gao L. Cui Y. N. Yang H. L. Silva N. D. G. D. (2020). Aliphatic suberin confers salt tolerance to Arabidopsis by limiting Na + influx, K + efflux and water backflow. *Plant Soil.* 448 603–620. 10.1007/s11104-020-04464-w

[B51] Wang Y. Stevanato P. Lv C. Li R. Geng G. (2019). Comparative physiological and proteomic analysis of two sugar beet genotypes with contrasting salt tolerance. *J. Agric. Food Chem.* 67 6056–6073. 10.1021/acs.jafc.9b00244 31070911

[B52] Xu J. Li H.-D. Chen L.-Q. Wang Y. Liu L.-L. He L. (2006). A protein kinase, interacting with two calcineurin B-like proteins, regulates K^+^ transporter AKT1 in *Arabidopsis*. *Cell* 125 1347–1360. 10.1016/j.cell.2006.06.011 16814720

[B53] Yao Y. Ding J. L. Zhang F. Wang G. Jiang H. N. (2013). Monitoring of soil salinization in Northern Tarim Basin, Xinjiang of China in dry and wet seasons based on remote sensing. *Chinese J. Appl. Ecol.* 24 3213–3220. 24564152

[B54] Zahra M. Soheil K. Reza R. M. (2017). Salinity induced changes in water relations, oxidative damage and morpho-physiological adaptations of pistachio genotypes in soilless culture. *Acta Agric. Slovenica* 109:291 10.14720/aas.2017.109.2.12

