# Supplementary material for: Overexpression of β-Ketoacyl-CoA Synthase From Vitis vinifera L. Improves Salt Tolerance in Arabidopsis thaliana
Source: Front Plant Sci. 2020 Nov 12;11:564385. doi: 10.3389/fpls.2020.564385 (PMC7688582; doi:10.3389/fpls.2020.564385)
Supplement: Supplementary file 1 [file Data_Sheet_1.docx]

Supporting Information:

**Fig. S1 Relative expression levels of genes related to suberin in 1103P (A) and Crimson Seedless (B).**

**
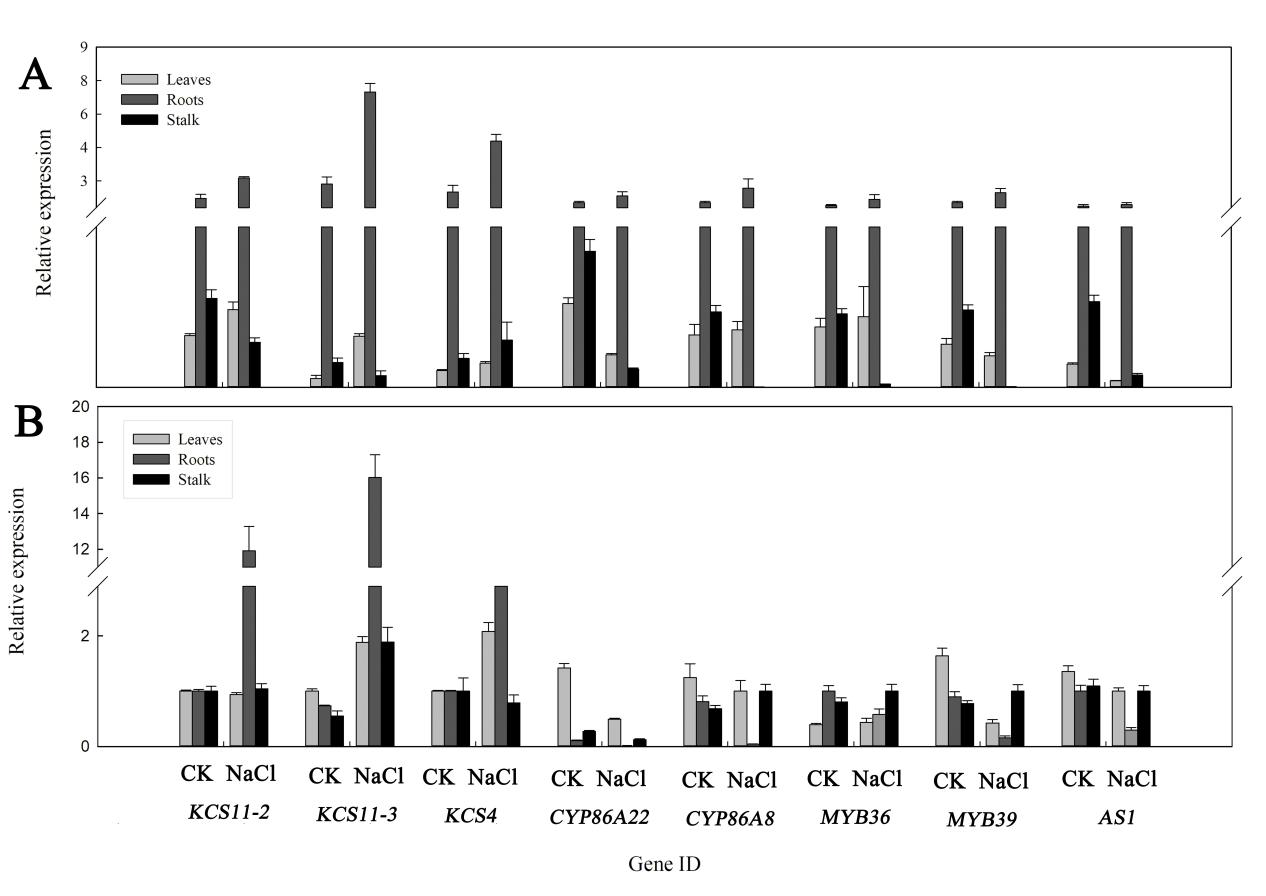
**

**Fig. S2 The nucleic acid sequences alignment of *VvKCS11* from1103P and Crimson Seedless**  (A); amino acid sequences alignment of VvKCS11 from 1103P and Crimson Seedless (B)


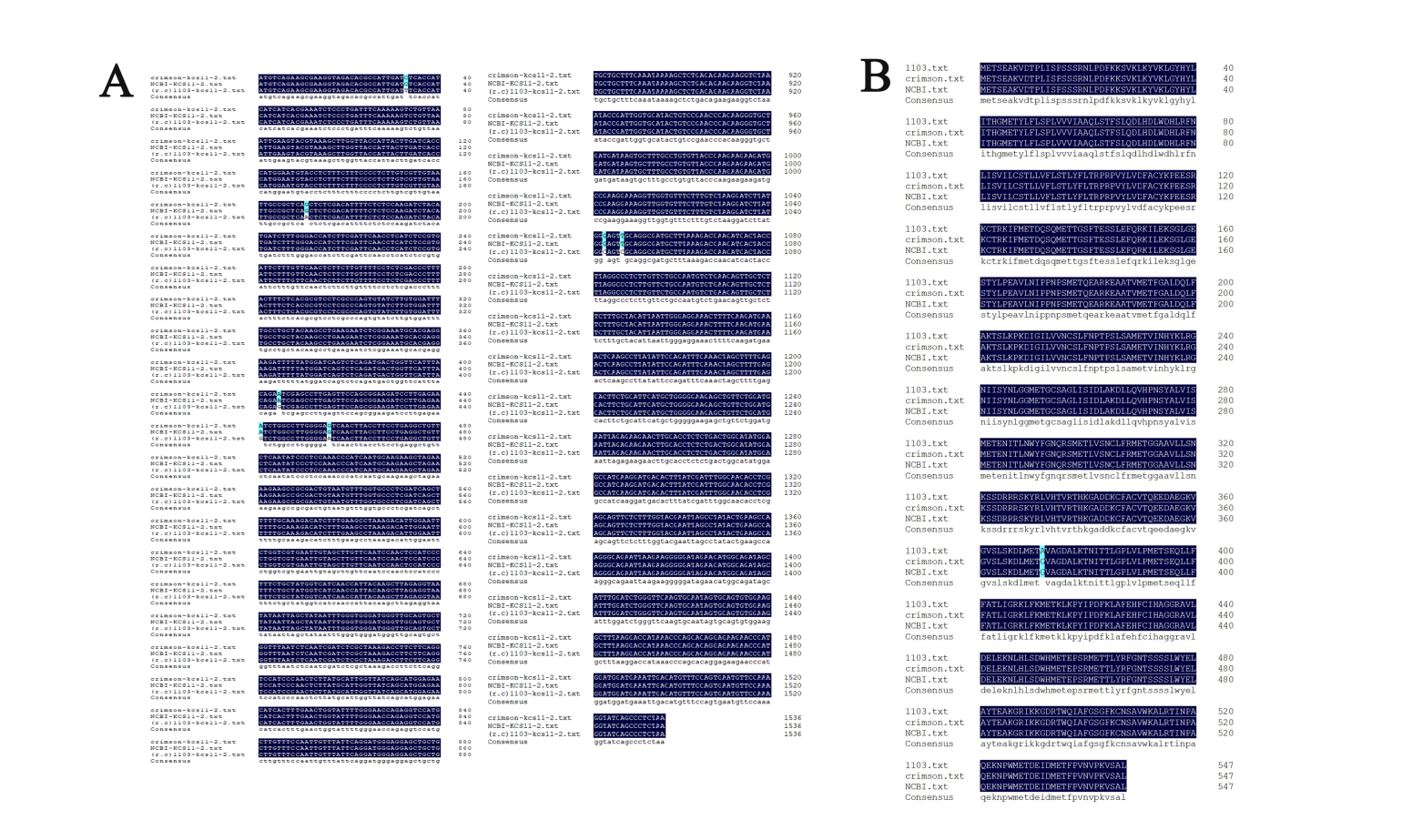


**Fig. S3** VvKCS11 gene of Crimson Seedless amino acid sequences（A）

Structural domain prediction of Crimson Seedless VvKCS11 protein (B)


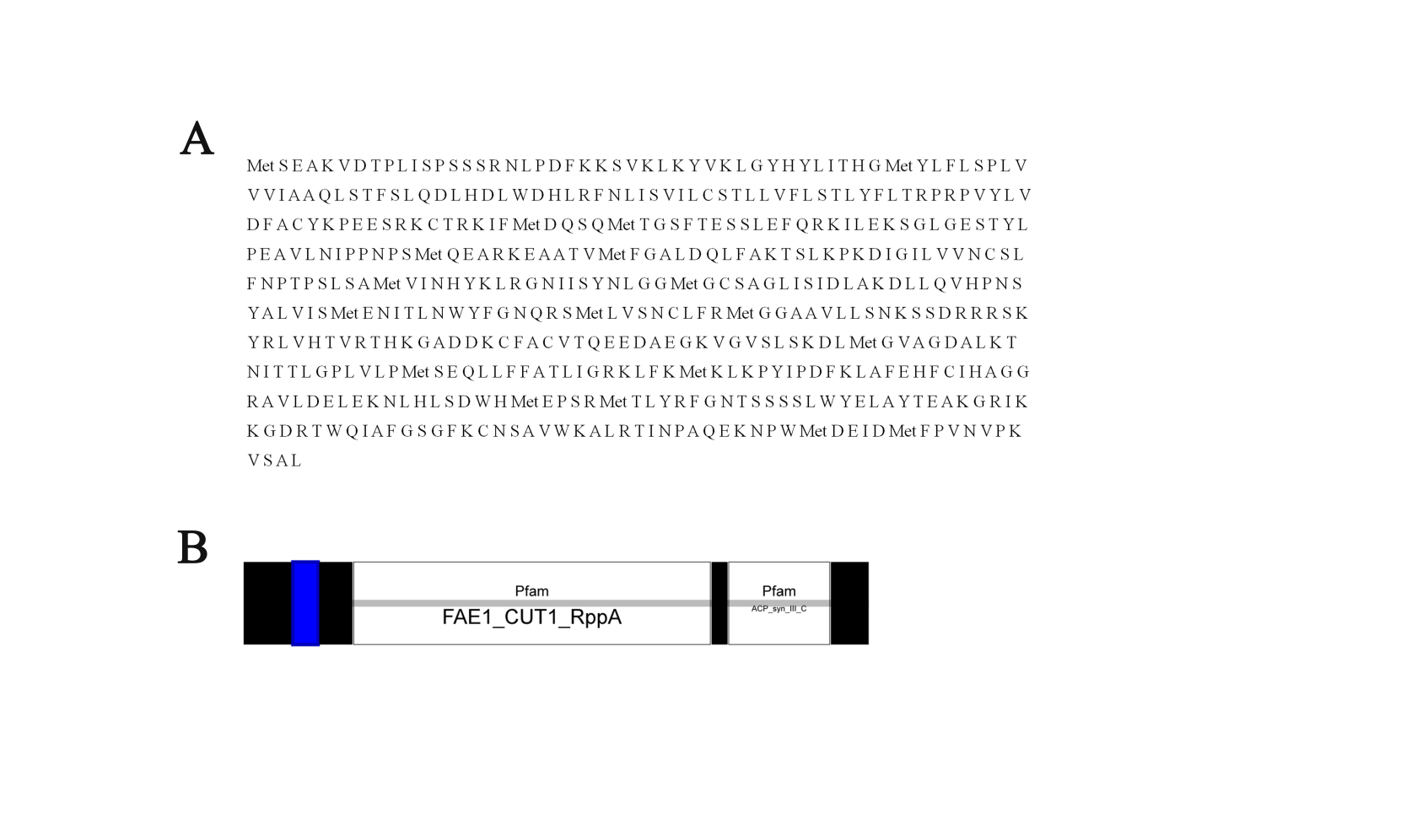


**Fig. S4 Evolutionary relationships of amino acid residue sequences of the VvKCS11**

**
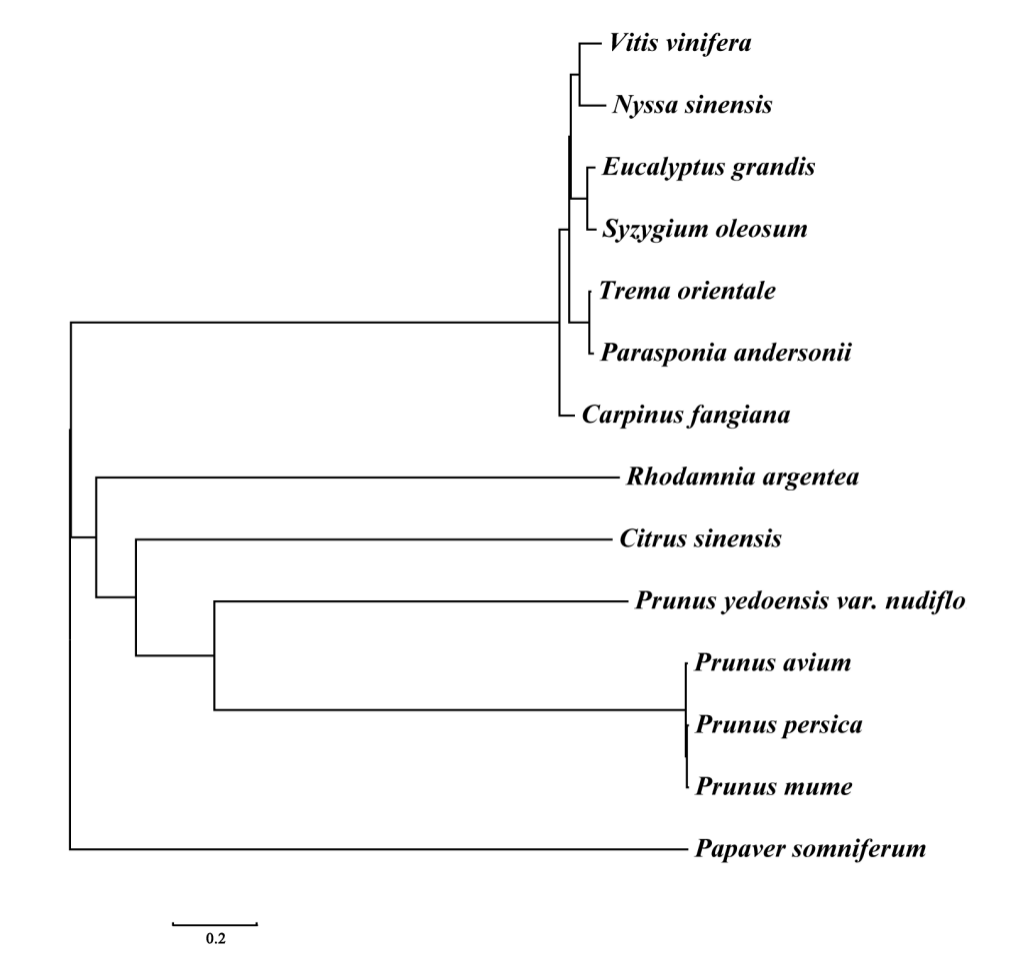
**

**Fig. S5** **Identification of *Arabidopsis* overexpression lines.** Total RNA was isolatedfrom leaves of *Arabidopsis* seedlings in culture. Kana screening in overexpressing lines of *Arabidopsis* (A); genomic DNA PCR of overexpression lines (B)


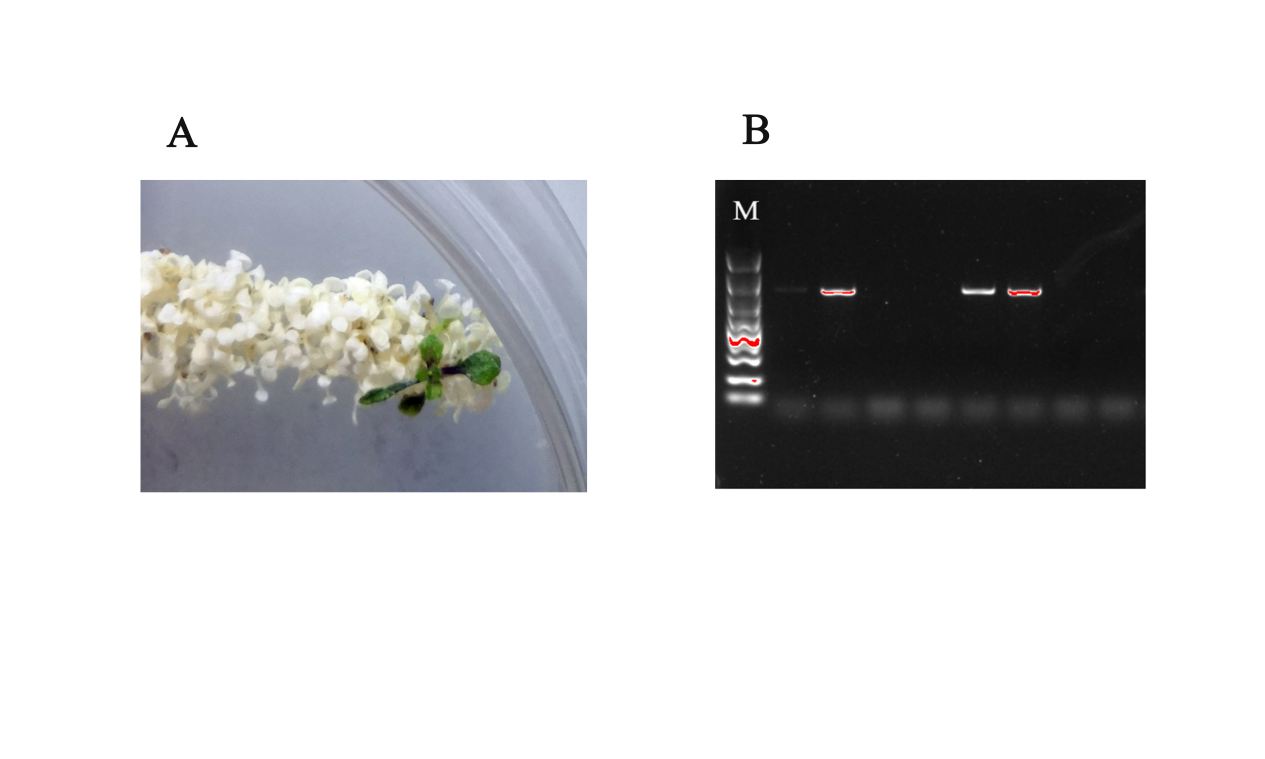


**A**

**Fig. S6 Relative expression levels of *VvKCS11* overexpression lines.**

**
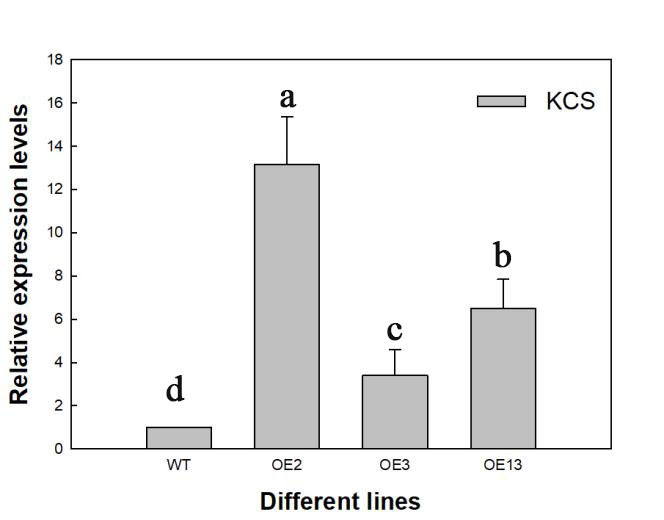
**

**Fig. S7 Identification of T-DNA insertion *Arabidopsis* mutant.** The T-DNA is forward inserted at the intron of atkcs11(At2G26640 ) genomic locus. T-DNA insertion was confirmed using PCR with the indicated primer sets, and the M refers to the 5000 bp marker.Lanes 1-5:LP+RP; Lanes6-10:LBB1+RP(A). Atkcs11 transcripts were determined by qPCR (B).


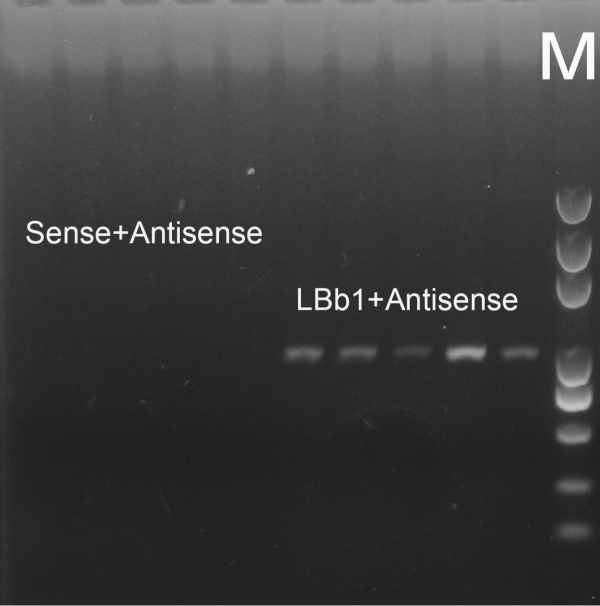


**Fig. S8** T**he germination rate of WT, overexpression lines of *Arabidopsis* under different NaCl concentrations.** The germination rate after senven days (**A**); The germination energy after senven days (**B**); The germination index after seven days(**C**).

**
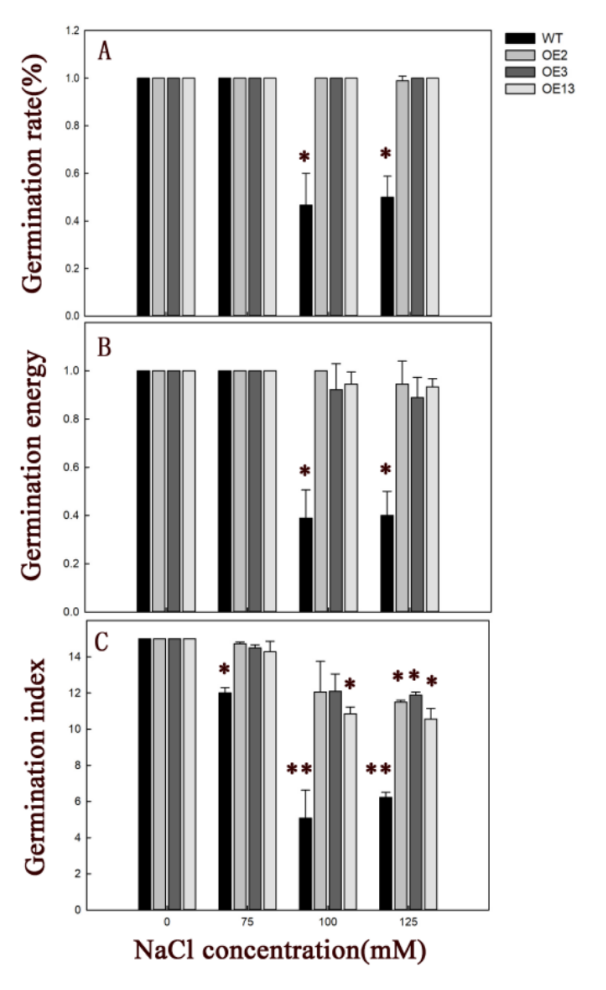
**

**Table S1 Genes and primer pairs for real-time quantitative PCR**

| **GeneID** | **accession** | **Sense primer （ 5’- 3’）** | **Antisense primer （ 5’- 3’）** |
| --- | --- | --- | --- |
| XM 002276970.3 | CYP86B1 | GCGTCTATAATATACCGTTA | GCGTCTATAATATACCGTTA |
| XM 0022838993 | KCS11-1 | GCATCCATTGTTACTTGA | GCATCCATTGTTACTTGA |
| XM 002284950.4 | KCS11-2 | GAAGCAGATGGAAGATAGC | AGTAACGAAGACAGAACCT |
| XM 002284715.4 | KCS11-3 | GTTCTGATGATAAGTGTT | CAAGGTAGTGATATTAGTC |
| XM 002282367.3 | KCS 4 | TACAGAGCGTCAACCTAA | TCAAGATTCCGATCATAAGAG |
| XM 002275770.3 | CYP86A22 | CACTAAGGTCAATGGTAA | ATGGCATCATATTCACAA |
| XM 002275079.3 | CYP86A8 | TTCCTCAACCAATCTCATT | AAGCCTCTGTCTATCTCT |
| XM 002270730.3 | MYB36 | ACAAGACTGAAGAAGAAG | GCCTGTGAATATGGATTA |
| XM 003633958. 3 | MYB39 | GACATCTCCAATCCTTCA | GTCACTTGCTTCATCATC |
| XM 002266391.3 | transcripton factorAS1 | CCACAGTTAGCACATCAA | CAGAGGAGAATGAACAAGTAA |
|  | VvACTIN | GAGATTCCGTTGTCCAGAAGTC | CAATGTTGCCATAGAGGTCCTT |

**Table S2 Genes and primer pairs for real-time quantitative PCR**

| Gene ID | name | Sense primer （5’--3’） | Anti-sense primer （5’--3’） |
| --- | --- | --- | --- |
| AT2G26650 | *atAKT1* | ACCGATAACATTGTGAACGCATTC | AGAAGGAACCAAGAACGCAAGTA |
| AT5G47100 | *AtCBL9* | TTGAGGACGCAGATGTGGAT | GGAGACATACCTGAGATACGGAA |
| AT1G30270 | *GAtCIPK23* | AGAAGTGGAGCAGAAGCAGTT | AGAACCTCGCAACACGCATA |
| AT2G01980 | *AtSOS1* | CTGGAGGAAGCGACCGATT | GAGAAGAGCGACAGTGTAAGGAA |
| AT5G27150 | *NHX1* | GCTTCTGTGGTTGCGTTGAA | AGGCGGTGATGGATTCGTT |
| At2g26640 | *KCS11* | TCAAGAAGAGGATTCCGCAAGT | AGAGCATCACCCGCAACA |
| At5g43760 | *KCS20* | ACAAACGCCGAGCCAAAC | GAGGAGAGGAAGGAGGAGGAT |
| At1g04220 | *KCS2* | ACAACTCGTCCTCGCAGAAT | TGAAGATACCGACACGCTGAG |
| AT4G19960 | *KUP9* | AACCACAACCACAACAACAACAT | ATCCGTCTCCTCCTCCTCTAC |
| AT4G10310.1 | *.HKT1* | CCTCATAACAAGATTAGA | TAGTAACAAGATGGTAAC |
| AT4G13420 | *HAK5* | TAATGTTGGTTATCTGGAA | CGTTATCGTAAGTGGTAA |
| AT1G06970 | *CHX14* | TTCAATCTCCTTATCATCA | TTACTCTTGCTTCTGTAG |
|  | *AtACTIN* | CTCCTTTGTTGCTGTTGACTAC | GCACAATGTTACCGTACAGATC |
